# Anticancer drugs-related QTc prolongation, torsade de pointes and sudden death: current evidence and future research perspectives

**DOI:** 10.18632/oncotarget.25008

**Published:** 2018-05-22

**Authors:** Jialin Duan, Jingwen Tao, Maocai Zhai, Chengpeng Li, Ning Zhou, Jiagao Lv, Lin Wang, Li Lin, Rong Bai

**Affiliations:** ^1^ Division of Cardiology, Department of Internal Medicine, Tongji Hospital, Tongji Medical College, Huazhong University of Science and Technology, Wuhan, P.R. China; ^2^ Department of Cardiology, Wuhan Hospital of Integrated Traditional Chinese and Western Medicine, Wuhan, P.R. China; ^3^ Department of Cardiology, An Zhen Hospital, Capital Medical University, Beijing, P.R. China; ^4^ Texas Cardiac Arrhythmia Institute at St. David’s Medical Center, Austin, TX, USA

**Keywords:** anticancer therapy, QT interval prolongation, torsade de pointes, molecularly targeted drugs

## Abstract

Anticancer drugs may have proarrhythmic effects including drug-induced QT interval prolongation, which is of particular importance because it can lead to a fatal polymorphic ventricular tachycardia termed torsade de pointes (TdP). QT interval prolongation and TdP are rare life-threatening untoward effects of anticancer therapy, particularly with arsenic trioxides and anthracyclines, and even some novel molecular targeted drugs touted as ‘tumor specific’. Several factors that affect myocardial repolarization can further increase the risk of TdP. This article reviews the mechanism of QT interval prolongation, risk factors for TdP and the QT toxicity of anticancer drugs as well as its management. Specific attention should be paid to high-risk populations such as patients with underlying heart diseases, electrolyte imbalance and bradycardia. To minimize the occurrence of QT interval prolongation and TdP, it is advisable to conduct a careful risk factor assessment before antitumor therapy. To this end, several new biomarkers have been introduced to predict TdP triggering and recent studies have pointed out the potential clinical relevance of genetic testing.

## INTRODUCTION

Cancer treatment has greatly benefited from recent developments in drug therapy. However, anticancer drugs despite their increased target specificity are not without toxicities, and among cardiac ones, prolongation of the QT interval is particularly concerning [1]. Many drugs affect heart repolarization and prolong the QT interval, thereby increasing the risk for torsades de pointes (TdP), a lethal ventricular arrhythmia, and sudden cardiac death. QT-interval prolongation and TdP not uncommonly underlie drug withdrawal from the market [2].

**Figure 1:**
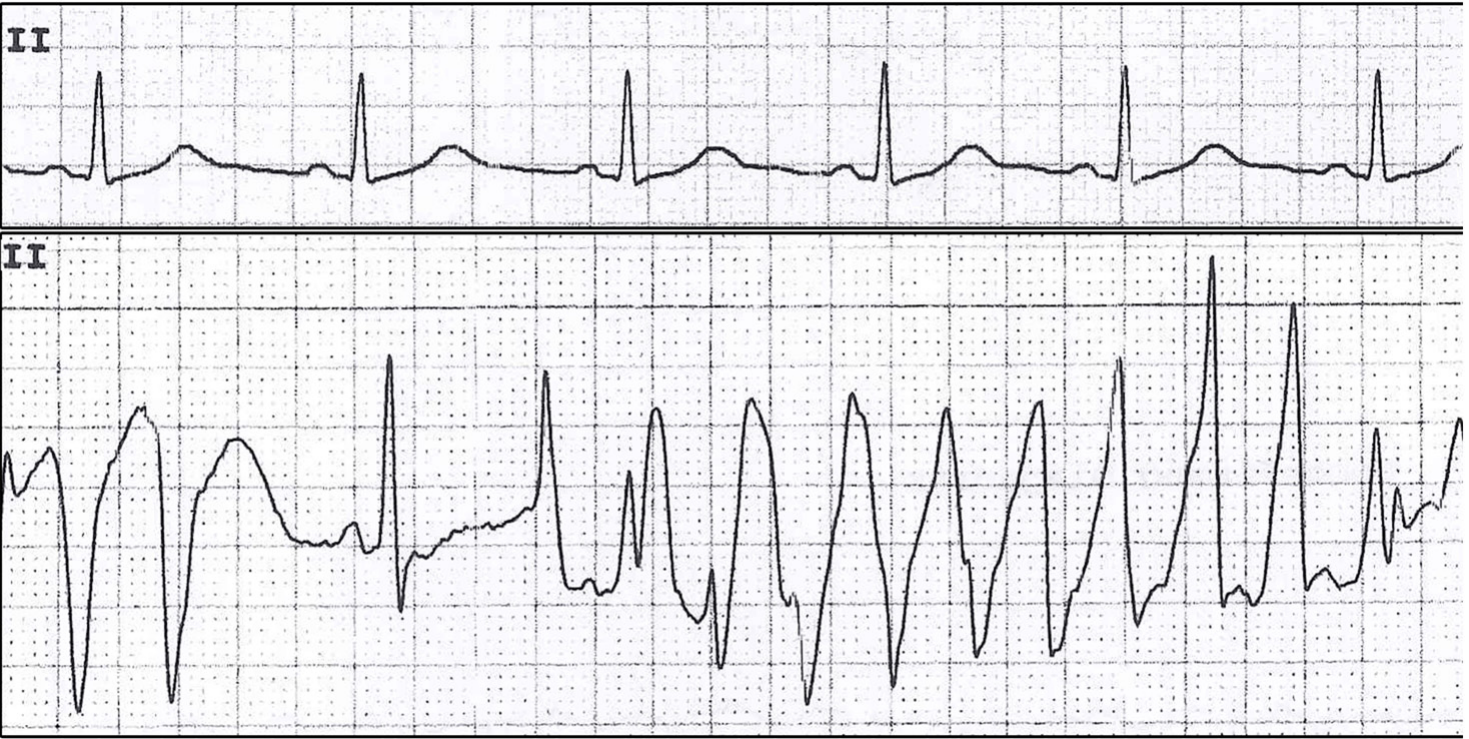
TdP in a patient with distant metastatic (M1) gastric adenocarcinoma undergoing oxaliplatin treatment

The term torsades de pointes (Figure 1) was first introduced by Dessertenne in 1966 as “a polymorphic ventricular tachycardia where QRS complexes twist around an isoelectric line” on the surface electrocardiogram (ECG) [3, 4]. Although polymorphic ventricular tachycardia with normal QT interval is also called TdP, its treatment is very different and most scholars stress that TdP only refers to polymorphic ventricular tachycardia with prolonged QT interval [5].

## QT INTERVAL MEASUREMENT

**Figure 2:**
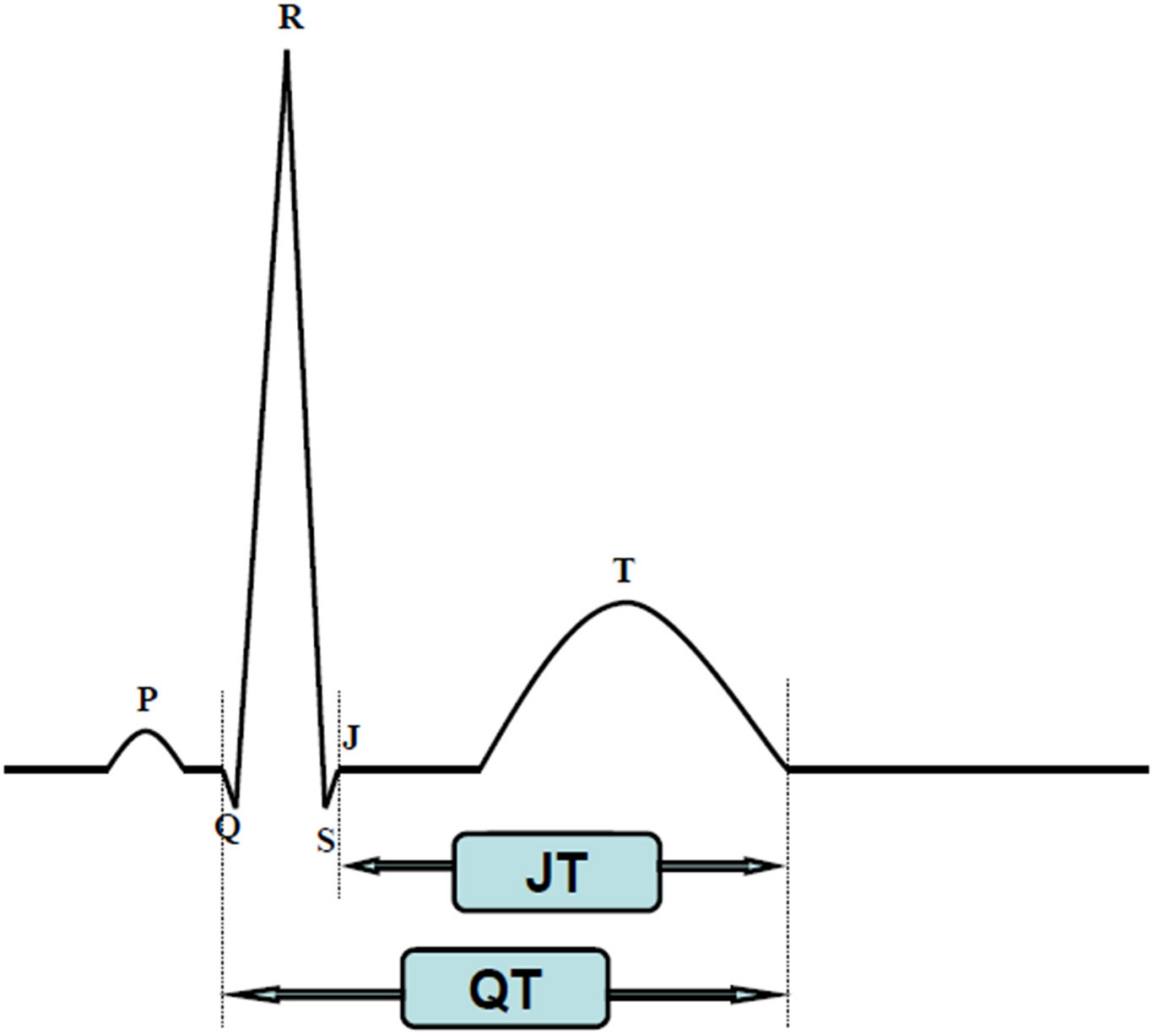
QT and JT interval measurement with ECG

The QT interval as measured on an ECG is measured from the beginning of the QRS complex to the end of the T wave, reflecting the depolarization and repolarization of both left and right ventricles. Usually, the QT interval is measured in lead II [12] and should be determined as a mean value derived from at least 3–5 cardiac cycles [6]. The QRS duration also influences the QT interval when perturbed by pre-excitation, bundle branch block, or pacemaker implantation. In these situations, the JT interval may offer more information [7, 8, 9]. Figure 2 QT and JT interval measurement with ECG [9], although standards for the JT interval are unclear at this time. The QT interval is closely related to heart rates, so a correction of the QT interval for heart rates is necessary. Bazett’s formula (QT_C_=QT/RR^1/2^) and the Fridericia formula (QT_C_=QT/RR^1/3^) are widely used to correct the QT interval. Bazett’s formula is considered to be the standard for this type of measurement although it may exaggerate the QT interval at fast heart rates [10, 11]. The Fridericia formula has similar problems but is more accurate with tachycardia [10, 11], and for patients with atrial fibrillation, the Fridericia formula is preferred [13]. A QT_C_<450 ms in women and a QT_C_<430 ms in men is considered abnormal with a stable sinus rate. QT_C_ values from 450 to 470 ms in women and 430 to 450 ms in men are considered borderline [6]. Values of QT_C_>500 ms are considered strikingly abnormal because deadly arrhythmic events can occur at this value.

## MECHANISM OF DRUG-INDUCED QT PROLONGATION

The QT interval as measured by ECG represents the total duration of ventricular depolarization and repolarization. Generally, the process of ventricular depolarization is much shorter than that of repolarization. Hence, factors that prolong ventricular action potential duration, especially repolarization, prolong the QT interval. An increase in the inward current and a decrease in an outward current will delay action potential duration. Among the ion currents of the action potential, two delayed rectifier potassium current subtypes, I_Kr_ (rapid) and I_Ks_ (slow) are important to ventricular repolarization [14]. Blockade of I_Kr_ which is encoded by the human ether-a-go-go-related gene (HERG) of chromosome 7 is the most common target of drug-induced QT prolongation [15]. The HERG channel (the KCNH2 potassium voltage-gated channel) is responsible for most QT-associated drug toxicity. However, HERG channel blockade is not necessarily related to QT prolongation. For example, raloxifene inhibits cardiac delayed rectifier potassium currents but does not lead to QT prolongation [16], underscoring uncertainties regarding the mechanism of drug-induced QT prolongation.

**Figure 3:**
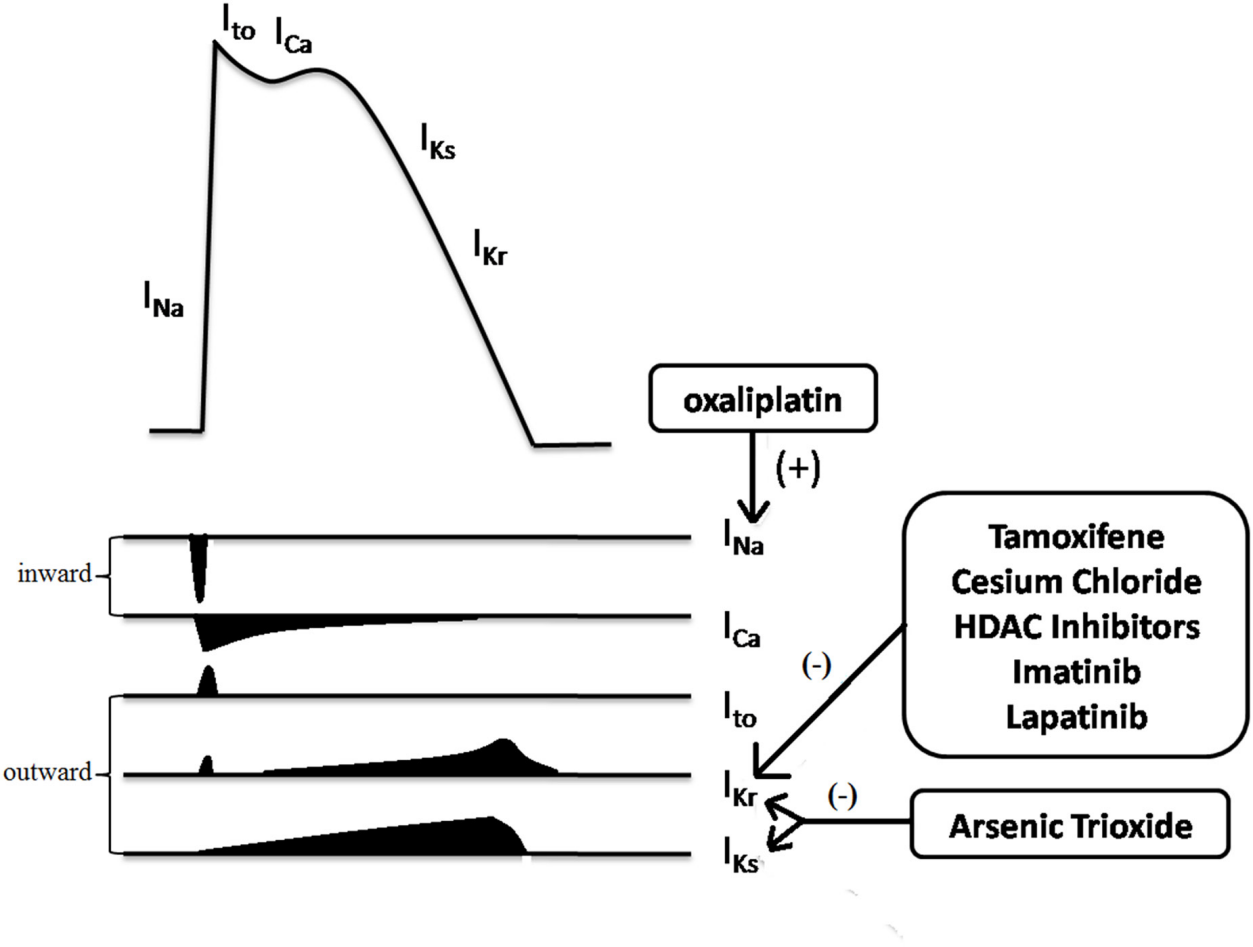
Multiple ion currents involved in the myocardial action potential Among them, I_Kr_ (HERG) is the most common target for anticancer drugs induced QT prolongation. Arsenic trioxide and oxaliplatin prolong the QT interval in unique ways. (+) represented for enhancing and (-) for inhibiting. I_to_, transient outward potassium current.

Figure 3 Arsenic trioxide blocks both I_Kr_ and I_Ks_ at clinically relevant concentrations and activates the I_K-ATP_ which maintains normal repolarization [17]. Oxaliplatin might prolong QT interval by increasing Na^+^ influx due to prolonged opening of Na^+^ channels [18]. However, the mechanisms underlying QT toxicity of many molecularly targeted cancer drugs need to be defined.

## RISK FACTORS AND PREMONITORY SIGNS OF TDP IN ECG

There are many risk factors (Table 1) for drug-induced long QT syndrome (diLQTS), including those that damage repolarization reserve, a term introduced by Roden [19] to reflect that normal cardiac repolarization depends on multiple ion currents which provide some redundancy or “reserve,” which protects against excessive QT prolongation by xenobiotics [19].

**Table 1:** Common clinical risk factors for drug-induced QT prolongation and TdP [19]

Female
Conditions predisposing to heightened QT prolongation and risk of arrhythmia
Heart disease
Congestive heart failure
Left-ventricular hypertrophy
Hours following conversion of atrial fibrillation to sinus rhythm
Congenital long-QT syndrome (may be clinically unrecognized)
Bradycardia and conduction disease
Increased drug bioavailability
Altered function of specific cytochrome P450 (CYP450) isoforms (for liver metabolized drugs) Genetic variants
Concomitant inhibitory drugs
Liver disease
Altered renal or liver function (for renally or hepatically excreted drugs)
Electrolyte imbalance
Hypokalaemia
Hypomagnesaemia
Hypocalcaemia (possible)
Administration of other drugs which might cause QT prolongation

**Figure 4:**
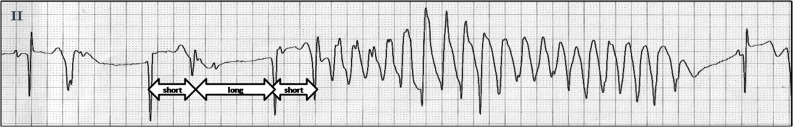
“Short-long-short” pattern prior to triggering a TdP

**Figure 5:**
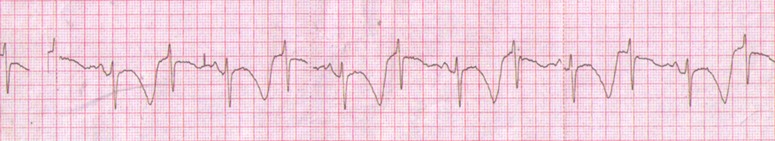
TWA in a patient with congenital long QT syndrome Minutes later, the patient developed TdP.

The ECG often features predictors of TdP. First, significant QT and QTc interval prolongation is essential for TdP—each 10-ms increase in QTc contributes a ∼5–7% exponential increase in TdP risk [20]. Almost all case reports of TdP caused by antineoplastic drugs occurred with a QTc>500 ms. Secondly, “short-long-short” patterns of R-R intervals (Figure 4) are often observed prior to TdP and this pattern consists of short-coupled premature ventricular complex (PVC) followed by a compensatory pause and then a second PVC that typically falls close to the peak of the T wave [5]. The first PVC is thought to increase the heterogeneity of repolarization of the next sinus beat and provoke TdP. Thirdly, T-wave alternans (TWA; Figure 5) which is a rare but dangerous ECG manifestation of TdP, manifests as a regular alternation of T-wave morphology with each beat [21] reflecting an instability of ventricular repolarization that predisposes to TdP.

## CLASSIC ANTINEOPLASTIC DRUGS

### Arsenic trioxide

Arsenic trioxide, which is widely used in patients with acute promyelocytic leukemia (APL), might cause prolongation of the QT interval and TdP at therapeutic doses [22]. Previous studies suggest that—compared with baseline—the QT_C_ interval was prolonged by 30–60 ms in 36.6% of treatment courses, and by more than 60 ms in 35.4% of patients [23]. The precise mechanism of arsenic trioxide-induced QT prolongation is complicated and the research results differ among species [24]. Furthermore, a recent study suggests that arsenic trioxide-induced cardiac fibrosis may involve in diLQTS [25]. In reported cases, arsenic trioxide-induced TdP frequently occurred several (usually more than 20) days after the start of therapy and other risk factors were present (Table 1) [26, 27], emphasizing the need for monitoring ECG and serum electrolyte data throughout therapy. Mild prolongation of the QT interval (440–500 ms) is common during treatment and does not limit the use of arsenic trioxide. Alpha lipoic acid has been reported to be protective against arsenic trioxide-induced QT interval prolongation in anesthetized guinea pigs [28], suggesting a potential approach for preventing arsenic trioxide-induced LQTS.

### Anthracyclines

Anthracyclines are effective for treating various tumors, including solid ones, and hematologic malignancies. However, the severe cardiotoxicity of anthracyclines has limited their use. QT interval prolongation and TdP are important signs of cardiotoxicity and there are several mechanisms of anthracycline-induced LQTS including effects on cardiomyocytes and ion currents. A plausible explanation for anthracycline-induced cardiomyocyte injury is the generation of reactive oxygen species (ROS) leading to oxidative stress [29, 30]. Ion channel effects also contribute to anthracycline-induced QT prolongation. Anthracycline treatment can increase sensitivity to the pro-arrhythmic potential of I_Kr_-blocking drugs and reduce redepolarization reserve [31] increasing patient susceptibility to drugs that promote TdP. Several cases of anthracycline-induced LQTS have been reported [32], mostly in female patients, one of whom died of TdP and ventricular fibrillation [33]. Most patients in the study were within the recommended safe dose range and the risk was conferred from weeks to years of anthracycline therapy. Risk factors such as hypokalemia and drug-drug interactions were common in these cases, specifically interactions between azole derivatives and arsenic trioxide. Dexrazoxane is FDA approved for prevention of anthracycline cardiotoxicity in women with breast cancer.

### Other classic drugs

Tamoxifen and its derivatives (4-hydroxytamoxifen) have been associated with QT interval alterations likely due to HERG potassium channel blockade [33]. 4-hydroxytamoxifen has been reported to inhibit K^+^ currents in mouse ventricular myocytes [34], and effects on calcium channels also contribute to QT-interval prolongation. A recent study suggests that high-dose tamoxifen decreases contraction amplitude, slows relaxation, and decreases the Ca^2+^ transient amplitude, which may explain tamoxifen-induced LQTS [35].

Platinum compounds have been associated with QT interval prolongation and TdP, especially oxaliplatin [36, 38, 39]. Oxaliplatin can increase Na^+^ influx by prolonging Na^+^ channel opening, thereby affecting cardiac repolarization [18]. Acetyl-L-carnitine, DL-α-lipoic acid and silymarin can ameliorate cardiotoxicity associated with cisplatin in rats [37], but further studies are needed before human use is considered.

Cesium chloride, an alternative or second-line treatment for several cancers, also has been reported to induce LQTS [41, 42, 43, 44]. TdP occurred several weeks to one year after cesium therapy in the presence of multiple risk factors (female gender, hypokalemia, and bradycardia). Cesium chloride blocks HERG potassium channels, prolonging the QT interval and inducing TdP [45]. Prussian blue has been reported to treat cesium chloride-induced LQTS [41].

5-fluorouracil, Cesium chloride, cyclophosphamide, paclitaxel and docetaxel are often associated with QT-interval prolongation [40], but this complication is infrequent and often due to high doses of the drugs or to drug-drug interactions.

## NOVEL ANTINEOPLASTIC DRUGS

### Trastuzumab

Trastuzumab, a monoclonal antibody against the Her2 signaling pathway, is widely used in Her2 metastatic breast carcinoma and acute trastuzumab infusion has been shown to have no effect on cardiac repolarization and the QT interval [47]. However, a recent study suggests that long-term (12 weeks) treatment with trastuzumab significantly prolonged QT intervals and increased QTd (QT dispersion) in patients with breast cancer [46]. Thus, chronic cardiomyocyte toxicity of trastruzumab may contribute to QT alterations. ACE inhibitors and beta-blockers can prevent trastuzumab-induced cardiotoxicity and recent clinic trials suggest that combined therapy with ACE inhibitors/angiotensin receptor blockers and beta-blockers may prevent trastuzumab-related cardiotoxicity [48].

### HDAC inhibitors

QT prolongation and TdP has also been observed in patients taking histone deacetylase with prolongation of the QT interval and sudden cardiac death in clinical trials. In a phase II study with 15 patients with metastatic neuroendocrine tumors, after an infusion of depsipeptide, one patient died suddenly and this was attributed to possible fatal ventricular arrhythmia. Two other patients had asymptomatic nonsustained ventricular tachycardia, and prolonged QT was noted in three patients [49]. Vorinostat, a HDAC inhibitor used to treat cutaneous T-cell lymphoma (CTCL), has been reported to induce TdP in a 49-year-old African American man [50]. Other HDAC inhibitors, such as romidepsin and LAQ824 have also been associated with QT-interval prolongation, but no fatal ventricular arrhythmia has been reported [51, 52]. HDAC inhibitor-induced QT interval prolongation and TdP is thought to occur via HERG channel blockade, but this is uncertain. The development of new HDAC inhibitors that are highly efficacious but with weak affinity for hERG may offer a way to prevent such adverse reactions.

### Tyrosine kinase inhibitors

The development of tyrosine kinase inhibitors (TKIs) is one of the most important advances in anticancer therapy these years. They have been widely used in solid tumors and hematologic malignancies. However, their use has been associated with underlying cardiac toxicities including hypertension, left ventricular dysfunction and QT prolongation [53]. Many TKIs, including crizotinib, ponatinib, nilotinib, cediranib, vandetanib, lapatinib, sunitinib, Bosutinib, Dasatinib, Lenvatinib, Osimertinib, Pazopanib, Ceritinib, imatinib, are associated with QT prolongation [54–61, 84–90]. Among them, Crizotinib deserves special mentioned. Crizotinib, an anaplastic lymphoma kinase (ALK) inhibitor, is FDA-approved for the treatment of non-small cell lung carcinoma (NSCLC) [62]. However, it has been associated with QT bradycardia and prolongation. In two clinical trials, 4/306 with QTc ≥500 ms and ΔQTc ≥60 ms in 10/289 were observed [63]. Besides, according to Ou’s research, there was an average decrease of 26.1 beats per minute (bpm) from pretreatment heart rate (HR) among all patients on crizotinib [64]. As mentioned, QT prolongation is more dangerous with bradycardia, so carefully monitoring in patients under the treatment of crizotinib is necessary. According to the formal studies, imatinib could preferentially block the open HERG channel, contributing to QT interval prolongation [65]. Lapatinib can block the HERG channel in transfected HEK293 cells and prolong the action potential duration [66], prolonging the QT interval. These data suggest that HERG channel blockade may explain QT prolongation, but more work is required.

### Rituximab

Rituximab is an anti-CD20 monoclonal antibody and effective for patients with lymphoid malignancies. However, cardiotoxicity of rituximab is likely under-reported. A case report regarding rituximab-induced polymorphic ventricular tachycardia [67] suggests that a female patient with malignant lymphoma who had undergone atrioventricular nodal ablation and pacemaker implantation, developed syncope due to polymorphic ventricular tachycardia during an initial rituximab infusion. Unfortunately, the ECG was not timely, and the arrhythmia was recorded by intracardiac electrogram from pacemaker interrogation, complicating the recognition of TdP. The QT interval was prolonged according to the ECG recorded later, underscoring the possibility that TdP should not be excluded. Other cardiac adverse reactions from rituximab, including acute coronary syndromes and complete atrioventricular block were observed [68, 69, 70], but how these occurred is unclear.

### Combretastatin A4 phosphate

Combretastatin A4 phosphate (CA4P) is a vascular-disrupting agent that targets existing tumor blood vessels can causes tumor necrosis. Adverse reactions after intravenous infusion of CA4P have been documented in a pilot study as was elevated blood pressure (46.7%), QTc prolongation (23.3%), elevated temperature (13.3%), and headache (10%), as well as by nausea and eye infection(6.7%) [71]. In a single-dose phase I study of patients with advanced cancer, significant increases in QTc intervals were observed at 3 or 4 h after CA4P infusion [72]. Two cases of CA4P-induced Takotsubo cardiomyopathy and QT interval prolongation were also reported [73] and for these two cases, elderly females who received combretastatin suffered severe decreased left ventricular systolic function and significant wall-motion abnormalities, but acute coronary syndrome was excluded suggesting that this acute myocardial injury might be associated with combretastatin.

### Other molecularly targeted drugs

Vemurafenib, a BRAF inhibitor, is effective for treating patients with BRAF V600E mutation-positive inoperable and metastatic melanoma but this drug is associated with QT prolongation. In a single-arm, open-label, expanded access study of vemurafenib among 374 patients, 2 patients were discontinued because of recurrent QT prolongation [74]. Thus, regular monitoring of ECGs is recommended during vemurafenib therapy [75]. Another BRAF inhibitor, Dabrafenib, has also been associated with QT prolongation [91].

Other molecular targeted drugs such as Eribulin (microtubule inhibitor), Bortezonib (proteasome inhibitor), are also associated with QT problems [76, 92, 93], but TdP has not been reported.

## DISCUSSION

Antineoplastic drugs that prolong the QT interval are listed as Table 2. Other common cardiotoxicities of anticancer drugs include left ventricular dysfunction and myocardial ischemia. Anthracyclines, anti-Her2 agents and anti-VEGF treatments were commonly associated with acute or chronic heart failure during anticancer therapy [94]. Takotsubo syndrome of cardiomyopathy (ballooning of left ventricle) was observed in two men treated with bevacizumab for metastatic cancer [95]. The myocardial ischemia most often associated with 5-FU or its fluoropyrimidine prodrug capecitabine is coronary vasospasm [96]. Bevacizumab can also lead to arterial thromboembolic events, including myocardial infarction in 0.6---1.5% of patients [97].

**Table 2:** Antineoplastic drugs that prolong the QT interval

*Drugs*	*Effect of QT*	*TdP*	*References*
**Classic drugs**			
*Arsenic Trioxide*	37% with 30–60 ms↑, 35% with > than 60 ms↑	**+**	[23, 26, 27]
*Anthracycline*	9.56 ms↑in doxorubicin therapy	**+**	[32]
*Tamoxifen*	Dose related	possible	[34]
*Platinum compounds*	6.25% in cisplatin treatment, no data for oxaliplatin treatment	**+** (oxaliplatin)	[36, 38, 39]
*5-fluorouracil (5-FU)*	Significant increases in QTmax and QTd were observed as early as 24 h after 5-FU treatment	**−**	[40]
*Cesium chloride*	QT prolongation usually occurs with total cesium intakes of 6 g/day	**+**	[41–44]
**Molecularly-targeted drugs**			
*Trastuzumab*	Prolongs QT interval with long-term treatment (12 weeks)	**−**	[46]
*HDAC inhibitors*	36% with vorinostat treatment	**+** (vorinostat and depsipeptide)	[49–52]
*Rituximab(possible)*	1 case report	possible	[67]
*TKIs*	Varieties of TKIs could lead to QT interval prolongation.	**−**	[54-61, 84-90]
*CA4P*	23.3% QTc prolongation	**−**	[71]
*BRAF inhibitors*	Vemurafenib and Dabrafenib	**−**	[74, 91]
Eribulin	a minor prolongation of QTc	**−**	[92]
Bortezonib	2 of 11 patients showed QT interval prolongation	**+**	[93]

Benefit/risk evaluations are warranted to improve safety of cancer therapy. Special attentions should be paid for patients undergoing chemotherapy with other factors that increase the risk of TdP, such as hypokaliemia, use of antibiotics that can prolong the QT (macrolides, quinolones) and use of psychotropic drugs. Because TdP is lethal, risk factors must be assessed and the risk must be managed where possible. Thus, more precise predictors for TdP are needed. New noninvasive biomarkers have been introduced to predict potential risks of drug-induced TdP. For example, T-wave morphology such as T(peak)-T(end) intervals, notched T wave, and giant T-U waves correlate with the risk of TdP. T(peak)-T(end) of 117 ms is also a reliable discriminator [77, 78]. Microvolt TWA may predict risks for sudden cardiac death [79] being useful in drug-induced LQTS. Index of cardiac electrophysiological balance (iCEB=QT/QRS) can help predict drug-induced cardiac arrhythmias including ventricular tachycardia/ventricular fibrillation (VT/VF) and TdP and may be more useful than current biomarkers [80]. Pharmacogenomics also may offer ways for predicting drug-induced LQTS. As genetic test has become less expensive in few years, more and more researches based on large populations have been investigated and more variants have been found. Both pharmacokinetics and pharmacodynamics are associated with diLQTS [81]. Interestingly, five to nineteen percent of patients with drug-induced torsade de pointes carry mutations in genes involved in congenital long QT syndrome [82]. Besides, genes that affect the drug metabolism such as cytochrome P450 substrates have also been proved with diLQTS [83]. Thus, a genetic background may be one of several factors for drug-induced LQTS. Also, specific drugs may have unique mechanisms of inducing TdP. Still, pharmacogenomics for predicting LQTS and TdP has the potential to be helpful in the future.

## Abbreviations

TdP: *torsade de pointes*
SNP: single nucleotide polymorphism
HERG: human ether-a-go-go-related gene
ECG: electrocardiogram
TWA: T wave alternans
PVC: premature ventricular complex
APL: acute promyelocytic leukemia
LQTS: long QT syndrome
ROS: reactive oxygen species
Top2β: topoisomerase-II β
EDTA: ethylenediaminetetraacetic acid
HDAC: histone deacetylase
CTCL: T-cell lymphoma
CA4P: Combretastatin A4 phosphate
iCEB: Index of cardiac electrophysiological balance
GWAS: Genome wide association studies
SJS/TEN: Stevens-Johnson syndrome/toxic epidermal necrolysis
diTdP: drug-induced torsade de pointes
